# Cardiovascular disease risk in female patients with mood disorder symptoms

**DOI:** 10.1192/j.eurpsy.2025.1056

**Published:** 2025-08-26

**Authors:** A. Hadj Ali, I. Chaari, N. Messedi, G. Chakchouk, F. Charfeddine, L. Aribi, J. Aloulou

**Affiliations:** 1 Psychiatric department “B”, Hedi Chaker University Hospital, Sfax, Tunisia

## Abstract

**Introduction:**

Patients with bipolar disorder (BD) and major depressive disorder (MDD) face an elevated risk of early mortality from various physical illnesses, particularly cardiovascular disease (CVD). However, it remains uncertain whether depressive or manic symptoms contribute more significantly to CVD outcomes.

**Objectives:**

This study aimed to assess the association between manic and depressive symptoms and long-term CVD risk factors.

**Methods:**

A retrospective study was conducted on patients hospitalized in the female unit of psychiatric department “B” in Sfax, Tunisia, between January and June 2023. Sociodemographic, clinical, and CVD risk factor data were collected from medical records to calculate Framingham Scores. The relationships between mood symptoms (depressive and manic) and Framingham scores, as well as individual CVD risk factors (lipids, blood pressure, BMI, smoking, and fasting glucose), were analyzed.

**Results:**

The study included 50 female patients with a mean age of 45 years (SD = 9.9). Of the patients, 18% were diagnosed with MDD, while 82% were diagnosed with BD; 64% were admitted for manic symptoms, and 36% for depressive symptoms. Clinical obesity was observed in 64% of patients, with 60% diagnosed with hyperlipidemia, 34% with hypertension, and 26% with type 2 diabetes. Notably, 12% of patients had a Framingham score of ≥ 10%, indicating a moderate to high 10-year risk of CVD. Bivariate analysis revealed that patients with manic symptoms had a significantly higher CVD risk than those with depressive symptoms (p=0.034). Manic symptoms were also significantly associated with elevated BMI (p=0.034), fasting glucose (p=0.039), and blood pressure (p<0.001).

**Conclusions:**

This study demonstrates a strong association between manic symptoms and long-term CVD risk in patients with mood disorders, particularly with elevated blood pressure, glucose levels, and BMI. These findings underscore the importance of specific monitoring and intervention programs for CVD risk factors in patients with mood disorders, especially those with BD.

**Disclosure of Interest:**

None Declared

